# Shared CSF Biomarker Profile in Idiopathic Normal Pressure Hydrocephalus and Subcortical Small Vessel Disease

**DOI:** 10.3389/fneur.2022.839307

**Published:** 2022-03-03

**Authors:** Anna Jeppsson, Maria Bjerke, Per Hellström, Kaj Blennow, Henrik Zetterberg, Petronella Kettunen, Carsten Wikkelsø, Anders Wallin, Mats Tullberg

**Affiliations:** ^1^Hydrocephalus Research Unit, Institute of Neuroscience and Physiology, The Sahlgrenska Academy, University of Gothenburg, Gothenburg, Sweden; ^2^Department of Psychiatry and Neurochemistry, Institute of Neuroscience and Physiology, The Sahlgrenska Academy, University of Gothenburg, Mölndal, Sweden; ^3^Clinical Neurochemistry Laboratory, Sahlgrenska University Hospital, Mölndal, Sweden; ^4^Clinical Neurochemistry Laboratory, Department of Clinical Chemistry, Universitair Ziekenhuis Brussel and Center for Neurosciences, Vrije Universiteit Brussel, Brussels, Belgium; ^5^Department of Biomedical Sciences, Institute Born-Bunge, University of Antwerp, Antwerp, Belgium; ^6^Department of Neurodegenerative Disease, UCL Institute of Neurology, Queen Square, London, United Kingdom; ^7^UK Dementia Research Institute at UCL, London, United Kingdom; ^8^Hong Kong Center for Neurodegenerative Diseases, Hong Kong, Hong Kong SAR, China

**Keywords:** biomarkers, cerebrospinal fluid, cerebral small vessel disease, hydrocephalus, normal pressure

## Abstract

**Introduction:**

In this study, we examine similarities and differences between 52 patients with idiopathic normal pressure hydrocephalus (iNPH) and 17 patients with subcortical small vessel disease (SSVD), in comparison to 28 healthy controls (HCs) by a panel of cerebrospinal fluid (CSF) biomarkers.

**Methods:**

We analyzed soluble amyloid precursor protein alpha (sAPPα) and beta (sAPPβ), Aβ isoforms −38, −40, and −42, neurofilament light protein (NFL), glial fibrillary acidic protein (GFAP), myelin basic protein (MBP), matrix metalloproteinases (MMP −1, −2, −3, −9, and −10), and tissue inhibitors of metalloproteinase 1 (TIMP1). Radiological signs of white matter damage were scored using the age-related white matter changes (ARWMC) scale.

**Results:**

All amyloid fragments were reduced in iNPH and SSVD (*p* < 0.05), although more in iNPH than in SSVD in comparison to HC. iNPH and SSVD showed comparable elevations of NFL, MBP, and GFAP (*p* < 0.05). MMPs were similar in all three groups except for MMP-10, which was increased in iNPH and SSVD. Patients with iNPH had larger ventricles and fewer WMCs than patients with SSVD.

**Conclusion:**

The results indicate that patients with iNPH and SSVD share common features of subcortical neuronal degeneration, demyelination, and astroglial response. The reduction in all APP-derived proteins characterizing iNPH patients is also present, indicating that SSVD encompasses similar pathophysiological phenomena as iNPH.

## Introduction

Idiopathic normal pressure hydrocephalus (iNPH) is a potentially reversible disorder and one of the very few treatable forms of dementia as shunt surgery improves around 80% of the patients (1, 2). The prevalence may be as high as 0.5–2.9% in people aged 65 or above (3–5). However, only 20–30% of patients are treated (5–8), largely due to underdiagnosis. There are no specific diagnostic markers available, diagnosis is presently based on clinical and radiological assessments in combination with cerebrospinal fluid (CSF) dynamic tests (9). Patients with iNPH exhibit impaired gait, balance and motor performances, reduced bladder control, and a fronto-subcortical cognitive and conative impairment with reduced volition, executive dysfunction, and memory loss (10). MRI studies have revealed that, apart from the hallmark of ventricular enlargement, periventricular and deep white matter hyperintensities (PVH and DWMH, respectively), markers of small vessel disease, are common in iNPH (11). iNPH patients with PVH and DWMH have been shown to respond well to shunting (12), but some studies have found the improvement of patients with signs of cerebrovascular disease (CVD) to be less pronounced than in those without (13, 14).

Patients with subcortical small vessel disease (SSVD), sometimes also referred to as Binswanger's disease, exhibit a fronto-subcortical cognitive impairment similar to iNPH and may show similar patterns of gait, balance, and urinary dysfunction (15). Furthermore, PVH and DWMH are hallmarks of SSVD, and enlargement of the brain ventricular system may be present, at least in later stages of the disease, partly due to brain atrophy. Adding to the complexity, it has been suggested that patients fulfilling the diagnostic criteria for SSVD can improve after shunt surgery (16). Moreover, a negative CSF tap test cannot be used to exclude patients with iNPH from shunt surgery (17, 18). Hence, to distinguish between iNPH and SSVD and to identify patients that might experience symptom relief following shunt surgery constitutes a major challenge.

Several studies have reported on both MRI and CSF biomarkers of possible differential diagnostic value for iNPH and SSVD. However, none of these have proven useful in the clinical setting (11, 19–23). A number of CSF biomarkers have been introduced to assess different aspects of neurodegeneration, for example, in Alzheimer's disease (AD) [for a review see ref. (24)]. Putative CSF biomarkers reflecting brain changes in SSVD have been reviewed by Wallin et al. (25). We have reported that a combination of CSF biomarkers reflecting amyloid metabolism (where in iNPH all APP fragments are reduced), cortical neuronal degeneration, and astrocyte activation could separate iNPH from movement and cognitive disorders (26), such as vascular dementia, with good sensitivity and specificity, and is thought to distinguish the pathophysiology in iNPH from these disorders.

To analyze similarities and differences, the biomarkers chosen in this study were soluble amyloid precursor protein alpha (sAPPα) and beta (sAPPβ), Aβ isoforms −38, −40, and −42 (Aβ38, Aβ40, and Aβ42, reflecting APP metabolism), neurofilament light protein (NFL, reflecting subcortical neural degeneration), glial fibrillary acidic protein (GFAP, reflecting astroglial response), myelin basic protein (MBP, reflecting demyelination), matrix metalloproteinases (MMP −1, −2, −3, −9, and −10, reflecting subcortical tissue remodeling), and tissue inhibitors of metalloproteinase 1 (TIMP1).

The aim of this study was to explore differences and similarities in CSF biomarkers between iNPH and SSVD. As the subcortical picture is overlapping in iNPH and SSVD, we included a biomarker panel reflecting subcortical damage and remodeling, comprising biomarkers reflecting amyloid pathology, subcortical neuronal degeneration, myelin damage, astroglial response, and extracellular matrix remodeling in search for pathophysiological similarities and differences. CSF from healthy controls (HCs) was examined as a reference.

## Materials and Methods

### Subjects

The study included 52 patients diagnosed with iNPH (aged 72; 68–79 (median; IQR) years; 29 men and 23 women), 17 patients diagnosed with SSVD (aged 72; 66–76 years; 5 men and 12 women) and 28 HCs (aged 67; 66–71; 18 men and 10 women).

The patients with iNPH were diagnosed between 2007 and 2012 at the Hydrocephalus Research Unit, Sahlgrenska University Hospital, Gothenburg, Sweden according to international guidelines (9).

The patients with SSVD, recruited between 2001 and 2012, were part of the Gothenburg mild cognitive impairment (MCI) study, comprising middle-aged to young elderly individuals with self-observed or informant-reported cognitive decline assessed by the physician as significant and without an obvious underlying causes, such as brain tumor, subdural hematoma, and major stroke (27). SSVD was diagnosed using the Erkinjuntti criteria (28). More specifically, the patients were required to have white matter changes [WMCs; mild, moderate, or severe according to Fazekas classification (28)] and predominant fronto-subcortical symptoms, such as mental slowness, executive dysfunction, and extrapyramidal motor signs, without pronounced memory loss. If WMCs were only mild, SSVD was diagnosed only if parietotemporal lobe syndromes, i.e., dysphasia, dyspraxia, dysgnosia, and loss of memory, were not marked (in which case mixed dementia, i.e., AD plus SSVD was indicated). Neither patients with mixed AD/SSVD dementia nor those with specific non-vascular neurodegenerative disorders with gait deficits, such as progressive supranuclear palsy and Parkinson's disease, were included in the study.

Healthy controls were primarily recruited through senior citizens organizations, e.g., at information meetings on dementia, and a small proportion were relatives of patients, also as part of the Gothenburg MCI study (27). To be regarded as healthy, the controls should not have experienced or exhibited any cognitive decline or have had diseases known to cause cognitive impairment at the time of inclusion.

### CSF Analyses

Lumbar CSF was obtained from the patients with iNPH prior to surgery and from SSVD and HC at the time of medical examination according to a standardized protocol. All lumbar punctures were performed in the morning to avoid any influence of possible diurnal fluctuations in biomarker levels and with the patient in a recumbent position. CSF samples were drawn at the lumbar vertebrae L3/L4 or L4/L5 interspace; the first portion of CSF was discarded to avoid blood contamination. The CSF was collected in polypropylene tubes and centrifuged at 2,000 × g at room temperature for 10 min. The ensuing supernatant was aliquoted in screw-cap polypropylene tubes and stored at −80°C pending biochemical analyses.

The concentrations of the amyloid-beta (Aβ) peptides −38, −40, and −42 and sAPP-α and -β, MMP −1, −2, −3, −9, −10, and TIMP1 were measured using single- or multiplex electrochemiluminescent immunoassays (Meso Scale Discovery, Rockville, MD, USA), following the instructions of the manufacturer with minor modifications. The neurofilament light chain (NFL) ELISA (NF-light^®^, UmanDiagnostics, Umeå, Sweden) analysis was performed according to a previously established protocol (29), with minor modifications. GFAP concentration was measured using a previously described in-house ELISA method (30). The analysis of MBP was performed with an ELISA (Active^®^ MBP, Diagnostic Systems Laboratories Inc., Webster, TX, USA), according to the instructions of the manufacturer. Intra- and inter-assay coefficients of variation were below 15% for all assays. All CSF analyses were performed batchwise in one round of experiments by laboratory technicians who were blinded to the clinical data.

### Radiological Evaluation

To stage subcortical damage by extent and distribution of WMCs, scans from iNPH patients and SSVD patients were evaluated by the age-related white matter changes (ARWMC) scale, which can be used for both CT and MRI images (28). WMCs were defined as bright lesions ≥5mm on transverse relaxation (T2), proton density (PD), or fluid-attenuated inversion recovery (FLAIR) sequences on MRI or as hypodense areas of ≥5mm on CT. Ratings were made in five different brain regions: frontal, parieto-occipital, temporal, basal ganglia, and infratentorial. In each region, the left and right hemispheres were rated separately, giving a total of ten regions. In each region, the ARWMC was rated from 0 to 3 (0 = no lesions; 1 = focal lesions; 2 = beginning confluence of lesions, and 3 = diffuse involvement of the entire region). Evans' index (EI) was determined on transaxial images as the ratio between the maximum diameter of the frontal horns and the maximum inner skull diameter and used as a measure of ventricular enlargement. All patients with iNPH and SSVD were rated by the same observer (AJ). MRI or CT images were lacking for one iNPH and three patients with SSVD.

### Statistics

Non-parametric statistical methods were used in all analyses due to non-symmetrical distributions and/or substantial differences in variances between groups (generally larger among patients than among controls). The Kruskal-Wallis one-way analysis of ranks (KW) was used to compare all three groups at once. The Wilcoxon-Mann-Whitney *U-*test was used for *post-hoc* analysis and for comparisons between pairs of groups. As the number of participants was few and the authors wished to avoid type 2 errors, no correction for the multiple comparisons was made. Alpha was set at *p* < 0.05. All analyses were performed in SPSS version 25.0 for Windows (IBM Corp, Armonk, NY, USA. Released 2014).

### Ethics

Participants (patients and/or their close relatives and HC) gave their written informed consent for participating in the study and for future results being published, in accordance with the World Medical Association Declaration of Helsinki. This study was approved by the Swedish Ethical Review Authority in Gothenburg, Sweden.

## Results

There was no difference in age or MMSE scores between patients with iNPH and SSVD, but the SSVD group had a higher percentage of women. Compared to controls, patients with iNPH were older, there were more women in the SSVD group and both groups had lower MMSE scores (Table 1).

**Table 1:** Age, sex, and mini-mental state examination (MMSE) in idiopathic normal pressure hydrocephalus (iNPH), subcortical small vessel disease (SSVD), and controls.

	**iNPH** ***n*** **= 52**	**SSVD** ***n*** **= 17**	**Controls *n* = 28**
Age, median (IQR)	72 (68–79)^#^	72 (66–76)	67 (66–71)
Female, *n* (%)	23 (44 %)	12 (71 %)	10 (36 %)
MMSE, median (IQR)	24 (22–27) ^###^	27 (25–28)^&&&^	30 (29–30)

*Age in years and mini-mental state examination (MMSE) are presented as median and interquartile range (IQR). Significance testing was made by Wilcoxon-Mann-Whitney U-test and shown as*

# *p < 0.05,*

### *p < 0.001 (iNPH vs. controls),*

&&& *p < 0.001 (SSVD vs. controls)*.

### CSF Biomarkers

**Figure 1:**
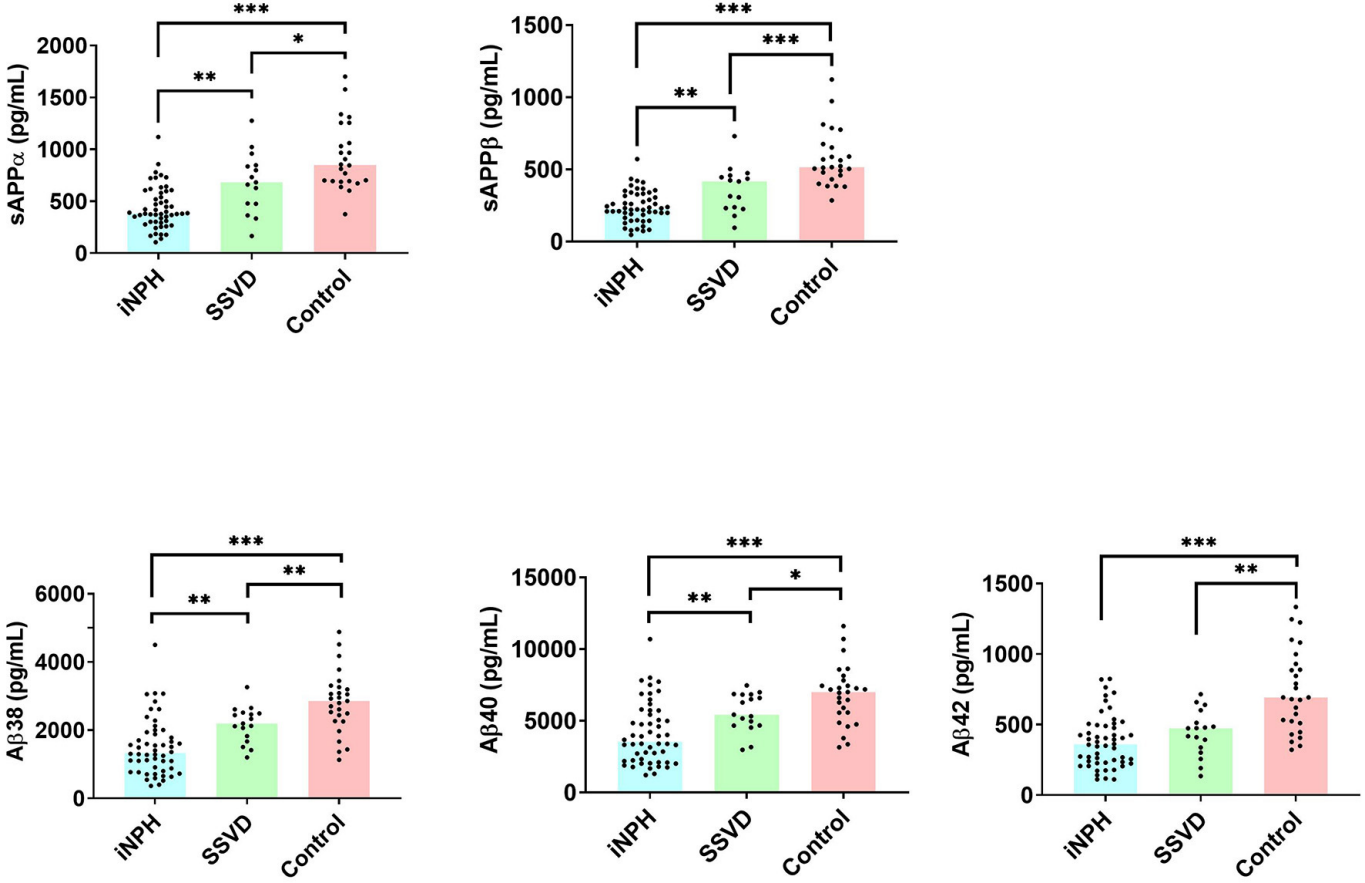
APP-derived cerebrospinal fluid (CSF) proteins in 52 patients with idiopathic normal pressure hydrocephalus (iNPH), 17 patients with subcortical small vessel disease (SSVD), and 28 controls. Concentration is plotted as individual values. Bars indicate median values. Only significant values are marked. iNPH, idiopathic normal pressure hydrocephalus; SSVD, subcortical small vessel disease; sAPP, soluble amyloid precursor protein; Aβ, amyloid-beta. ^*^p < 0.05, ^**^p < 0.01, ^***^p < 0.001.

**Figure 2:**
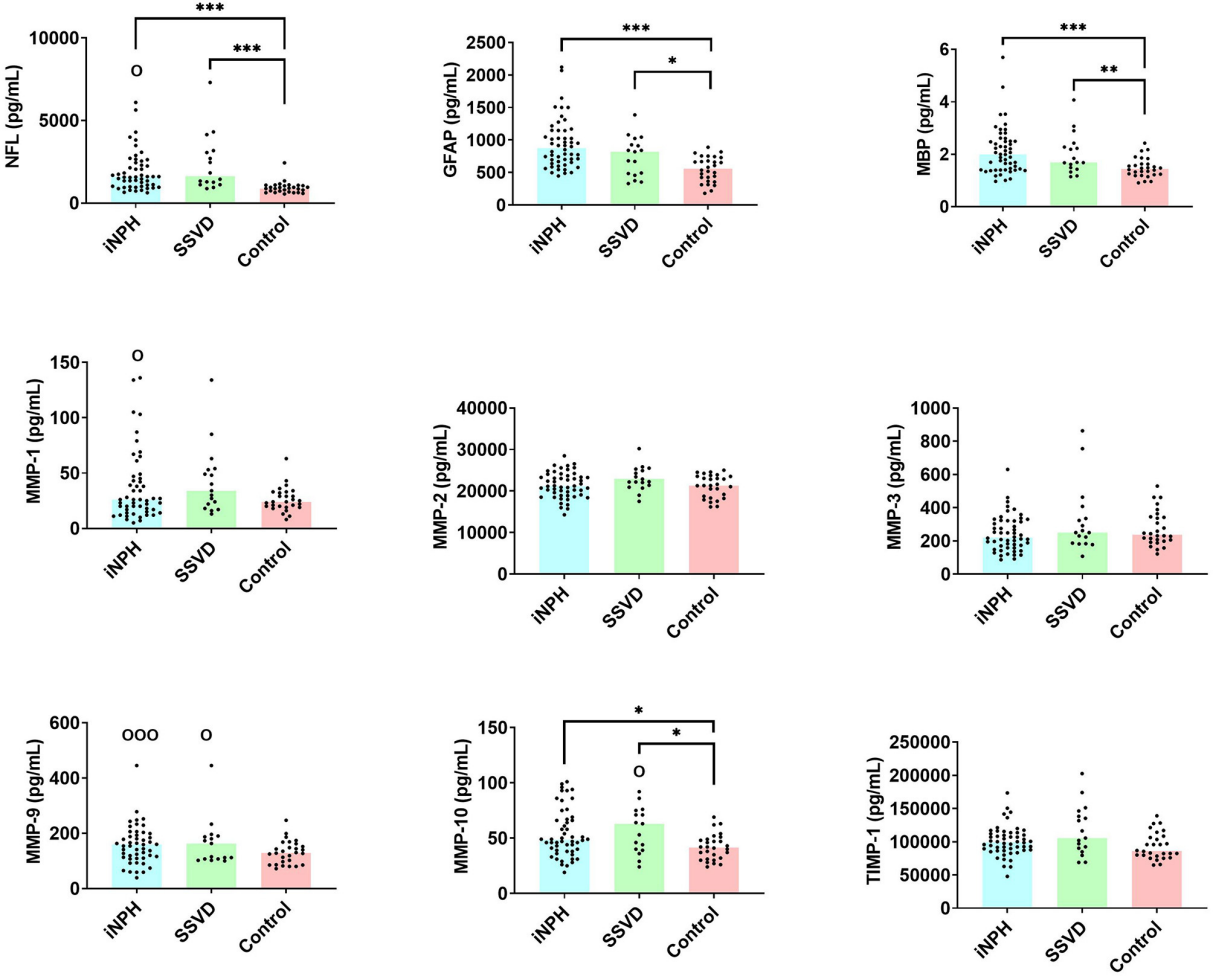
Cerebrospinal fluid (CSF) biomarkers of white matter damage, astroglial response, and extracellular matrix remodeling in 52 patients with iNPH, 17 with SSVD, and 28 controls. Concentration is plotted as individual values. Bars indicate median values. Only significant values are marked. iNPH, idiopathic normal pressure hydrocephalus; SSVD, subcortical small vessel disease; NFL, Neurofilament light protein; GFAP, glial fibrillary acidic protein; MBP, myelin basic protein; MMP, matrix metalloproteinases; TIMP, tissue inhibitors of metalloproteinases. O indicates values outside the axis.

All APP-derived proteins were lower in iNPH than in SSVD and controls and lower in SSVD patients than in controls. Markers of WMCs, subcortical neuronal degeneration (NFL), demyelination (MBP), and astroglial response (GFAP), were increased in iNPH and in SSVD compared to controls. Of the markers of extracellular matrix remodeling, MMP-10 was increased in iNPH and SSVD in comparison to controls Table 2, Figures 1, 2).

**Table 2:** Cerebrospinal fluid (CSF) biomarker concentrations in iNPH, SSVD, and healthy controls.

	**iNPH**	**SSVD**	**Controls**
	***n*** **= 52**	***n*** **= 17**	***n*** **=28**
sAPPα	384 (303–593)^###^, ^§§^	683 (475–847)^&^	850 (694–1207)
sAPPβ	227 (170–325)^###^,^§§^	417 (232–458)^&&&^	516 (446–664)
Aβ38	1,333 (823–1,928)^###^, ^§§^	2,196 (1,749–2,505)^&&^	2,855 (2,266–3,261)
Aβ40	3,541 (2,206–5,648)^###^, ^§§^	5,428 (4,678–6,838)^&^	7,009 (5,570–7,814)
Aβ42	361 (232–496)^###^	474 (320–558)^&&^	693 (510–931)
NFL	1,592 (1,012–2,519)^###^	1,638 (1,150–3,149)^&&&^	889 (694–1,072)
GFAP	876 (659–1,146)^###^	820 (472–976)^&^	559 (381–718)
MBP	1,997 (1,407–2,503)^###^	1,691 (1,461–2,351)^&&^	1,446 (1,228–1,632)
MMP−1	26 (16–47)	34 (20–54)	24 (19–33)
MMP-2	21,190 (18,965–23,600)	22,244 (21,146–25,104)	21,317 (18,423–23,549)
MMP-3	221 (162–322)	250 (186–372)	238 (201–344)
MMP-9	160 (114–205)	163 (107–193)	129 (89–160)
MMP-10	49 (38–67)^#^	63 (40–76)^&^	42 (31–49)
TIMP-1	99,329 (87,306–113,161)	105,464 (87,590–142,345)	86,094 (78,696–107,987)

*CSF biomarker concentrations in pg/ml shown as medians and interquartile ranges (IQR). Wilcoxon-Mann-Whitney U-test*.

# *p < 0.05, ^##^p < 0.01,*

### *p < 0.001 (iNPH vs. controls);*

§§ *p < 0.01, (iNPH vs. SSVD);*

& *p < 0.05,*

&& *p < 0.01,*

&&& *p < 0.001 (SSVD vs. controls). iNPH, idiopathic normal pressure hydrocephalus; SSVD, subcortical small vessel disease. sAPP, soluble amyloid precursor protein; Aβ, amyloid-beta; NFL, neurofilament light chain; GFAP, glial fibrillary acidic protein; MBP, myelin basic protein; MMP, matrix metalloproteinase; TIMP, tissue inhibitor of metalloproteinase*.

### Imaging Measures

On the group level, both iNPH and SSVD patients exhibited dilated ventricles, as indicated by an EI > 0.3, although patients with iNPH had more pronounced ventricular enlargement. Of the 14 patients with SSVD who were included in the radiological evaluation, 9 patients had an EI of 0.3 or above (64.3%). ARWMC scores were higher in SSVD than in patients with iNPH (Table 3).

**Table 3:** Age-related white matter changes (ARWMC) scores and Evans' Index in patients with iNPH and SSVD.

**Brain region**	**iNPH** ***n*** **= 51**	**SSVD** ***n*** **= 14**
Frontal		
R	1 (1–2)	2 (2–3)
L	1 (1–2)	2 (2–3)
Parietal-occipital		
R	1 (1–2)	2.5 (1–3)
L	1 (1–2)	2.5 (1–3)
Temporal		
R	0 (0–1)	0 0–0)
L	0 (0–1)	0 (0–0.25)
Basal ganglia		
R	0 (0–1)	1 (0–2.25)
L	0 (0–0)	1 (0–2.25)
Infratentorial		
R	0 (0–1)	1 (0–2)
L	0 (0–1)	0.5 (0–2)
**Total**	**7 (4–11)**	12 (8–22)^**^
Evans' Index	0.4 (0.37–0.44)	0.31 (0.27–0.38)^***^

*ARWMC scores are presented for each sub-region, right (R) and left (L) hemisphere separately, and as total scores. Values are given as median and interquartile ranges*.

** *p < 0.01,*

*** *p < 0.001*.

## Discussion

In this study, we show that all APP-derived proteins were lower in both iNPH and SSVD compared to controls, patients with iNPH exhibited a more pronounced reduction than patients with SSVD, while biomarkers related to white matter damage and astroglial response were increased in both disorders. Further, radiological WMCs were more pronounced in SSVD than in iNPH, whereas patients with INPH had more pronounced ventricular enlargement.

### APP-Derived Biomarkers

A reduction of all measured APP-derived proteins in CSF has been shown repeatedly in iNPH and thus, this study corroborates earlier results (31–35). It has also been shown in iNPH that CSF concentrations of amyloid precursor protein-like protein (APLP) and its derivatives, processed by the same enzymatic machinery, are not affected in the same manner as APP-derived proteins, which points to a specific disturbance of APP-metabolism in iNPH, possibly due to decreased periventricular metabolism (36). An additional proposed mechanism is a reduced clearance of these proteins from the interstitial fluid to the CSF (37). The pattern of reduction of all APP proteins in SSVD reported here is similar to that in iNPH, albeit deviating less from the concentrations in CSF of controls. Lower Aβ42 in iNPH than in SSVD has been reported previously (38).

### Biomarkers of Neuronal Degeneration, Myelin Damage, and Astroglial Response

Neurofilament protein is the dominant protein of the axonal skeleton and as a CSF biomarker, it reflects neuronal death and axonal degeneration (39, 40). In iNPH and SSVD, multiple studies have shown an increase of NFL in CSF, corroborating the results on NFL reported here (31, 34, 38, 41), the similarly increased NFL levels implying a comparable degree or mechanism of axonal degeneration in these disorders.

Increment of MBP, a constituent of the myelin sheath and marker of damage to oligodendroglia, reported here for iNPH and SSVD, is in line with the periventricular and deep white matter damage and has previously been shown to be increment in both iNPH (31) and SSVD (42). We also show that GFAP, indicative of reactive astrocytes and astroglial response, is increased in iNPH and SSVD patients alike, which has been reported earlier (19, 43, 44). Again, levels are similar, indicating similar degrees of astroglial response in these disorders. These similarities in white matter degeneration, hinted at by the NFL, MBP, and GFAP concentrations, are contradicted by the difference in radiological WMCs as indicated by the higher ARWMC score in patients with SSVD. These findings are in line with the findings of Abu-Rumeileh et al., who found no correlation between increased levels of CSF and ARWMC rating (34).

### Matrix Metalloproteinases

To our knowledge, CSF levels of MMPs or TIMPs have not earlier been reported in iNPH in relation to HC or SSVD. Other studies have shown that ECM proteins, MMPs, and their substrates increase iNPH following shunt surgery (45). MMPs are believed to be activated by ischemic conditions and to play a major role in neuro-inflammation by, e.g., disruption of the blood brain barrier (BBB) and degradation of substances in the extracellular matrix (ECM), promoting ECM turnover (46). Inhibition of the MMPs is primarily regulated by TIMPs that are also thought to have independent biological actions. Here, increased CSF levels of MMP-9 and TIMP1 (at trend level), together with increased NFL and MBP, are in agreement with earlier suggestions of what characterizes a subcortical CSF profile and with earlier findings in patients with SSVD (42). This again indicates corresponding pathophysiological processes in the two disorders, albeit more pronounced in iNPH. Although interesting from a pathophysiological standpoint, this study does not support the use of these MMPs for diagnosing iNPH.

Patients with SSVD had more abundant WMCs on MRI or CT than patients with iNPH, why we expected the CSF biomarkers reflecting white matter damage, NFL and MBP, to be higher in SSVD. This, however, was not the case. The reason for this discrepancy between the biochemical similarity and radiological differences could be a more active degeneration of white matter in iNPH and thus augmented leakage to the CSF, despite less radiological evidence at standard sequences in CT or MRI. Alternatively, the pathophysiology in iNPH is more dynamic, with potentially reversible functional changes, whereas SSVD is characterized by more static and irreversible changes of the white matter, which the CSF biomarker pattern might suggest. Another radiological approach, e.g., perfusion- or diffusion-weighted MR imaging, could probably give an even better estimate of qualitative differences in the PVH and DWMH.

A possible interpretation of our results is that SSVD is affected by the same pathophysiological process as iNPH although to a lesser degree. In earlier reports, we have proposed that SSVD with ventricular enlargement may represent one form of hydrocephalus (11) and that iNPH pathology may induce periventricular vascular changes (47) or, alternatively, that severe vascular pathology may cause a CSF dynamic disturbance in itself (16). We speculate that the ventricular dilatation in SSVD, with 64% showing EI > 0.3, could be both related to CVD-related atrophy and secondary to a CSF dynamic disturbance similar to that seen in iNPH.

Idiopathic normal pressure hydrocephalus is characterized by disturbed CSF dynamics. CSF is absorbed through the periventricular capillaries and perfusion is reduced in the periventricular tissue (48, 49) which in turn leads to oxygen deprivation of the vulnerable glial cells. In SSVD, the WMCs are regarded as secondary to an arteriolar dysfunction and the reduced ability of the vessels to supply the highly perfused subcortical tissue (50). Vascular risk factors, such as hypertension, diabetes, and hyperlipidemia, are important in both iNPH and SSVD (15, 51, 52) and are likely the causes of arteriolar dysregulation. The deep white matter is affected in both diseases as indicated by the ARWMC rating reported here, but the ventricles are, at a group level, larger in iNPH. The ventriculomegaly in iNPH is considered secondary to the CSF dynamics whereas the enlargement in SSVD is supposedly due to atrophy, with loss of white matter, but, as discussed earlier, a certain element of disturbed CSF flow could be expected in SSVD. Our results further enhance the similar affection of subcortical structures in the two disorders.

The overlap of clinical, radiological, and biochemical characteristics is considerable and stresses the delicate questions facing the diagnosing clinician. It has been shown that patients with iNPH and extensive WMC can respond to shunt treatment (16, 53). A double-blind placebo-controlled study showed that iNPH patients with the heavy burden of vascular pathology and a negative CSF tap test can benefit from shunt surgery (16). It has even been proposed that the iNPH state may induce periventricular vascular changes or, alternatively, that severe vascular pathology may cause a CSF dynamic disturbance (11, 47). Our results indicate that the overlap is also evident in CSF biomarkers reflecting subcortical damage. As such, these biomarkers do not seem to hold any clear differential diagnostic value.

The similarities point to the importance of considering a CSF dynamic disturbance also in patients with SSVD and enlarged ventricles. Therefore, the next step should be to further examine which patients with SSVD might be diagnosed with iNPH and considered eligible for shunt surgery. Further, in future studies, it would be of interest to prospectively study the development of iNPH and SSVD to gain an understanding of the progression of pathophysiological events in iNPH and SSVD, and how these events are reflected in the biomarker changes.

There are some limitations that need to be addressed. We have tried to select as clear-cut patient groups as possible, diagnosing patients at specialized clinics for iNPH, and SSVD using up-to-date diagnostic criteria. The diagnoses are, however, clinical and without post-mortem verification. Given the similarities of these disorders and potential overlap between diagnostic criteria, we cannot rule out the possibility that patients with SSVD with enlarged ventricles could have been diagnosed as iNPH or vice versa. Moreover, as patients with abnormal Aβ42/Aβ40 ratio were not sought for and excluded, the inclusion of some cases of comorbid iNPH or SSVD and preclinical AD cannot be fully ruled out. The patients included reflect the routine clinical setting and we believe that the results reported here are representative and mirror true pathophysiological characteristics of iNPH and SSVD. One limitation is the rather small group sizes, especially the SSVD group, and HC, which may have caused more subtle differences in biomarker concentrations to be undetected due to low statistical power. Studies comparing larger groups are therefore warranted.

Patients with iNPH and SSVD have parts of the cognitive profile in common. MMSE was used to grade the severity of cognitive decline but is a rather crude tool for cognitive staging, and therefore, might underestimate differences between the groups. The radiological rating of WMC was done using the ARWMC scale because some patients had undergone MRI and some CT. A weakness of this scale is that it does not differ between periventricular and deep white matter changes.

## Conclusion

By examining CSF biomarkers in patients with iNPH and SSVD and in HCs, we show that there are some similarities in the CSF biomarker pattern of iNPH and SSVD and that this pattern is different from that of HC. Patients with iNPH exhibit greater deviations from HC regarding APP-derived proteins than patients with SSVD, but these changes, along with increased markers of white matter damage and astroglial response, are also evident in patients with SSVD. Radiologically, patients with SSVD display more extensive WMCs. We argue that the underlying pathophysiology in iNPH and SSVD might share common features. The presumable overlaps and divergences in pathology deserve further investigation; expanding the knowledge of how vascular small vessel disease and CSF dynamic disturbances are interrelated might render idiopathic NPH less idiopathic.

## Data Availability Statement

The raw data supporting the conclusions of this article will be made available by the authors, without undue reservation.

## Ethics Statement

The studies involving human participants were reviewed and approved by the Swedish Ethical Review Authority in Gothenburg, Sweden. The patients/participants provided their written informed consent to participate in this study.

## Author Contributions

AJ participated in the design of the study, coordinated the study, statistical analysis, the interpretation of data, and drafted the manuscript for intellectual content. MB, KB, and HZ participated in the design of the study, laboratory analyses, interpretation of data, and revised the manuscript for intellectual content. PH and PK participated in the interpretation of data and drafted the manuscript for intellectual content. CW and MT participated in the design of the study, coordination of the study, interpretation of data, and drafted the manuscript for intellectual content. AW participated in the design of the study, interpretation of data, and drafted the manuscript for intellectual content. All authors read and approved the article for submission.

## Funding

This study was supported by unrestricted grants from Gothenburg Foundation for Neurological Research, the Edit Jacobson Foundation, the John and Britt Wennerström Foundation, the Per-Olof Ahl Foundation for research on vascular diseases of the brain, the Rune and Ulla Amlöv foundation, Konung Gustaf V: s and Drottning Victorias Frimurarstiftelse. Support was also given by grants from the Swedish state under the agreement between the Swedish Government and the country councils, the ALF agreement (2017-04961; ALFGBG-720121, −720661, −715986, −720931, −724331, and −932618). KB is supported by the Swedish Research Council (#2017-00915), the Alzheimer's Drug Discovery Foundation (ADDF), USA (#RDAPB-201809-2016615), the Swedish Alzheimer's Foundation (#AF-742881), Hjärnfonden, Sweden (#FO2017-0243), and European Union Joint Program for Neurodegenerative Disorders (JPND2019-466-236). HZ is a Wallenberg Scholar supported by grants from the Swedish Research Council (#2018-02532), the European Research Council (#681712), the Alzheimer's Drug Discovery Foundation (ADDF), USA (#201809-2016862), and the UK Dementia Research Institute at UCL. The funding agencies were not involved in the study design, in the collection, analysis, and the interpretation of data; in the writing of the report; or in the decision to submit the paper for publication.

## Conflict of Interest

KB has served as a consultant, at advisory boards, or at data monitoring committees for Abcam, Axon, Biogen, JOMDD/Shimadzu. Julius Clinical, Lilly, MagQu, Novartis, Roche Diagnostics, and Siemens Healthineers, and is a co-founder of Brain Biomarker Solutions in Gothenburg AB (BBS), which is a part of the GU Ventures Incubator Program. HZ has served at scientific advisory boards and/or as a consultant for Abbvie, Alector, Annexon, AZTherapies, CogRx, Denali, Eisai, Nervgen, Pinteon Therapeutics, Red Abbey Labs, Roche, Samumed, Siemens Healthineers, Triplet Therapeutics, and Wave, has given lectures in symposia sponsored by Cellectricon, Fujirebio, Alzecure, and Biogen, and is a co-founder of Brain Biomarker Solutions in Gothenburg AB (BBS), which is a part of the GU Ventures Incubator Program. CW receives an honorarium for lecturing by Codman, Integra. MT has received an honorarium for lecturing by Codman, Integra. The remaining authors declare that the research was conducted in the absence of any commercial or financial relationships that could be construed as a potential conflict of interest.

## Publisher's Note

All claims expressed in this article are solely those of the authors and do not necessarily represent those of their affiliated organizations, or those of the publisher, the editors and the reviewers. Any product that may be evaluated in this article, or claim that may be made by its manufacturer, is not guaranteed or endorsed by the publisher.
